# Interpreting the Australian Dietary Guideline to “Limit” into Practical and Personalised Advice

**DOI:** 10.3390/nu7032026

**Published:** 2015-03-20

**Authors:** Flavia Fayet-Moore, Suzanne Pearson

**Affiliations:** Nutrition Research Australia, Level 13 167 Macquarie St, Sydney 2000, Australia; E-Mail: suzanne@nraus.com

**Keywords:** dietary guidelines, discretionary foods, ready reckoner, dietary advice

## Abstract

Food-based dietary guidelines shift the focus from single nutrients to whole diet. Guideline 3 of the Australian Dietary Guidelines (ADG) recommends “limiting” discretionary foods and beverages (DF)—Those high in saturated fat, added sugars, salt, and/or alcohol. In Australia, DF contribute 35% of total energy intake. Using the ADG supporting documents, the aim of this study was to develop a food‑based educational toolkit to help translate guideline 3 and interpret portion size. The methodology used to produce the toolkit is presented here. “Additional energy allowance” is specific to gender, age, height and physical activity level, and can be met from core foods, unsaturated fats/oils/spreads and/or DF. To develop the toolkit, additional energy allowance was converted to serves equaling 600 kJ. Common DF were selected and serves were determined based on nutrient profile. Portion sizes were used to calculate number of DF serves. A consumer brochure consisting of DF, portion sizes and equivalent number of DF serves was developed. A healthcare professional guide outlines the methodology used. The toolkit was designed to assist dietitians and consumers to translate guideline 3 of the ADF and develop a personalized approach to include DF as part of the diet.

## 1. Introduction

Dietary guidelines provide health professionals, policy makers and the public with evidence-based recommendations that promote health and wellbeing and reduce chronic disease risk. They are developed to guide food choices to optimize nutrient intake and improve eating patterns. In recent years, there has been a shift away from nutrient-based recommendations to considering whole foods in the context of total diet and food intake patterns. Globally, food-based dietary guidelines (FBDG) have been promoted as an important part of national food and nutrition policies [1,2,3]. Dietary patterns proposed by FBDG help individuals meet their nutrient intakes by recommending intake of nutrient-dense foods and limiting intake of nutrient-poor foods. In order to be effective in optimizing health, there is a need for FBDG to be translated and personalized to facilitate compliance and ultimately behaviour change. Personalization of dietary advice can serve to empower individuals to make dietary changes relevant to them [4]. It is important that the behavior change be maintained. To implement FBDGs, the joint FAO/WHO consultation report recommends providing a qualitative version for the public and supporting quantitative materials aimed at dietitians and policy-makers [5].

FBDG typically include recommendations around nutrient-dense foods and beverages, *i.e.*, those that are high in fat, sugar, salt and alcohol. The general consensus is to “limit”, “avoid”, “reduce” or consume these foods and beverages “sometimes” or “occasionally”. For example, guideline 3 of the Australian dietary guidelines (ADG; Table 1) advises Australians to limit intake of foods containing saturated fat, added salt, added sugars and alcohol [6]. Similarly, Americans are told to consume fewer foods with sodium, saturated fats, trans fats, cholesterol, added sugars, and refined grains [7]; the Eat Well Plate of the UK suggests consuming just a small amount of high fat/sugar foods [8]; Canadians are guided to limit foods and beverages high in calories, fat, sugar or salt (sodium) [9]; and several European countries, including Switzerland, Austria and Luxemburg, recommend limiting intake of saturated fat, sugar and salt but do not quantify this recommendation [3]. While the advice is sound, its meaning can be lost in the absence of quantitative guidance. How exactly does one interpret terms like “limit”, “small amounts” and “sometimes” to translate them into meaningful, personalized advice?

**Table 1:** Guideline 3 of the 2013 Australian dietary guidelines [6].

Limit intake of foods containing saturated fat, added salt, added sugars and alcohol.
a. Limit intake of foods high in saturated fat such as many biscuits, cakes, pastries, pies, processed meats, commercial burgers, pizza, fried foods, potato chips, crisps and other savoury snacks.
● Replace high‑fat foods, which contain predominantly saturated fats such as butter, cream, cooking margarine, coconut and palm oil with foods, which contain predominantly polyunsaturated and monounsaturated fats such as oils, spreads, nut butters/pastes and avocado.
● Low-fat diets are not suitable for children under the age of 2 years.
b. Limit intake of foods and drinks containing added salt.
● Read labels to choose lower sodium options among similar foods.
● Do not add salt to foods in cooking or at the table.
c. Limit intake of foods and drinks containing added sugars such as confectionary, sugar-sweetened soft drinks and cordials, fruit drinks, vitamin waters, energy and sports drinks.
d. If you choose to drink alcohol, limit intake. For women who are pregnant, planning a pregnancy or breastfeeding, not drinking alcohol is the safest option.

In the U.S., these nutrient-dense foods and beverages are considered foods and food components to reduce [7]; in Australia they are termed “discretionary foods” (DF) as they do not fit into the Five Food Groups, or core food groups of the ADG and are not needed to meet nutrient requirements. DF are not an essential part of dietary patterns. Nevertheless, as acknowledged by the ADG, “they [DF] can contribute to the overall enjoyment of eating, often in the context of social activities and family or cultural celebrations.” Being part of the Australian diet, the ADG advise DF can be included occasionally if energy needs allow but that they should always be considered “extras” in the context of energy requirements and when selecting a healthy eating pattern [10]. Consumers are encouraged to check the nutrition information panel found on packaged foods and beverages to determine what amount of food contains 600 kJ and examples of DF are offered. However, there is no specific guidance on how many choices can be included in the diet of an individual based on their age, gender, height and physical activity level nor clear cut-offs or guidelines for what constitutes a DF.

There is a gap between dietary recommendations and actual consumer behaviour and compliance with the guidelines is generally poor. According to the most recent data from the 2011 to 2012 National Nutrition and Physical Activity Survey, Australians are consuming less vegetables, fruits, grains and dairy than recommended, while DF contribute 35% and 39% of the total daily energy intake for adults (≥19 year) and children and adolescents (<18 year), respectively [11]. Comparatively, DF contributed 36% of daily energy intake of adults and 41% of energy intake of children and adolescents [12] in the nationally representative 1995 National Nutrition Survey (NNS) and 35% [13] in the 2007 Australian National Children’s Nutrition and Physical Activity Survey for children. Similarly, the majority of the US population did not meet recommendations for all of the nutrient-rich food groups, except total grains and meat and beans. Concomitantly, overconsumption of energy from solid fats, added sugars, and alcoholic beverages was ubiquitous. Over 80% of adults 71-years and over, and 90% of all other sex-age groups had intakes exceeding the discretionary calorie allowances [14].

A dietary guideline implementation strategy is as equally important as the development of the evidence-base that inform the guidelines. The need for effective communications to assist in translating the recommendations into practical, actionable advice is widely acknowledged and has been included as part of the release of guidelines globally [2,5,15,16].

There is a need to develop food-based resources to assist in translating dietary recommendations into practice. With approximately 60% of Australian adults overweight and more than 25% obese [11], this is especially important when it comes to nutrient-dense DF. Therefore, the aim of this research was to develop a food-based educational toolkit to help dietitians and consumers translate guideline 3 of the ADG. Specifically, to calculate the maximum number of DF serves that can be included as part of the diet based on gender, age, height and physical activity level, and to provide guidance on the number of serves of common DF and their portion size.

## 2. Experimental Section

### 2.1. Resources Used

The ADG [6] and the following supporting documents were used to develop the toolkit:

(i) *A Modelling System to Inform the Revision of the Australian Guide to Healthy Eating (Modelling System)* [17]—A technical document that translates the nutrient reference values into dietary models. It describes the amounts of various foods needed to meet the estimated nutrient requirements of groups of Australian individuals of different age, gender and physical activity level.
(ii) *Eat for Health Educator Guide* [10]—Developed for dietitians, nutritionists, primary and secondary school teachers and other health educators with the aim of discussing food choices that minimise the risk of developing diet‑related conditions and to contribute to overall health in the long term.
(iii) *The Eat for Health website* [18]—The online platform for the ADG.

### 2.2. Determining how DF Fit into the Diet: The “Additional Serves” Toolkit

To determine where DF fit into the diet, the “*Foundation Diets*” and “*Total Diets*” dietary models of the Modelling System were used. These diets demonstrate that while nutritional needs are met through the whole diet and not by single foods, the combination of foods is critical. The *Foundation Diets* are the dietary patterns that meet the nutrient and energy requirements for the smallest, youngest and least active individuals in each age and gender group accounting for chronic disease, food supply and social and cultural constraints. In addition to including foods from the Five Food groups (grain foods; vegetables and legumes/beans; fruit; lean meats and poultry, fish, eggs, tofu and nuts; milk, yoghurt, cheese and alternatives), or core foods of the ADG, an allowance for unsaturated fats, oils and spreads was used in the development of the *Foundation Diets*. The *Total Diets* provide a range of flexible options to add to the *Foundation Diets* to meet the higher energy requirements of people of varying body size and higher physical activity levels (PAL). Thus, the *Total Diets* includes the *Foundation Diets* and an “additional energy allowance”. DF were considered in modelling *Total Diets* so that any “additional energy allowance” can be consumed from foods of the Five Food groups, unsaturated fats, oils and spreads and/or DF (Table 2).

**Table 2:** Rationale for determining how “discretionary foods” (DF) fit into the diet.

Total Diet = Foundation Diet + Additional Energy Allowance	
❖ Meets energy & nutrient needs	✧ Age
	✧ Height
	✧ PAL
Five food groups	Five food groups
Unsaturated fats/oils/spread	Unsaturated fats/oils/spreads
	AND/OR
	✓ Discretionary foods

### 2.3. Ready Reckoner

In order to develop the toolkit, the additional energy allowance had to be translated into additional serves. A ready reckoner (RR) was developed based on age, gender, height (for adults only) and physical activity level specific to additional serves so that dietitians and nutritionists could quickly determine maximum daily DF intake.

One additional serve was defined as the kilojoule content of a DF serve from the ADG (*i.e.*, 600 kJ; 143 kcal). Additional serves were calculated by dividing the additional energy allowance provided in the Educator Guide (Appendix) by 600 kJ (143 kcal) and rounding to the nearest whole number. For example, 1200 kJ (287 kcal) equals 2 serves.

Additional serves are based on gender, age, height (for adults only) and physical activity levels. The age and height categories for the RR were obtained from the Educator Guide. Adults were grouped into the following age bands: 19–30 years, 31–50 years, 51–70 years and >70 years. Height bands ranged from 160 cm to 190 cm for males and 150 cm to 180 cm for females. For children, an energy (kJ) value was provided for each single age between 2- and 18-years, independent of height. As there was a discrepancy in the terminology used to describe physical activity levels between the ADG, the Educator Guide and the Modelling System, a consistent definition based on those described on the Eat for Health website was established. Physical activity categories from the additional energy allowance tables were converted to those described online (Table 3).

**Table 3:** Physical activity categories for the ready reckoner (RR).

Educators Guide Additional Energy Tables	Modelling System Physical Activity Levels (PAL)	RR Physical Activity Categories	RR Physical Activity Definitions
Inactive	Very sedentary (PAL 1.4)	Sedentary	Sedentary work and no strenuous leisure activities (e.g., an office worker who drives to and from work and spends most of their leisure time sitting or standing).
Light	Light to moderate (PAL 1.5–1.7)	Light	Mostly sedentary work with little or no strenuous leisure activity (e.g., an office worker who only occasionally exercises (once or twice a week), lab assistants or drivers).
Moderate		Moderate	Moderately active work, predominantly standing or walking (e.g., waiters, shop assistants or teachers).
High	Heavy occupational or high activity (PAL 2.0)	Vigorous	Heavy activity (e.g., tradesperson or high performance athlete).

### 2.4. Consumer Brochure

A consumer-friendly brochure was developed to be used with the RR. The brochure outlines the number of additional serves in common DF and their equivalent portion size. To develop the consumer brochure it was necessary to define DF, group examples of popular DFs into simple, consumer-friendly categories and determine their typical serving sizes.

The ADG provides examples of DF, but no clear cut-offs for foods containing saturated fat, added salt and/or added sugars are described; except for alcohol, which is easily identified.

The Modelling System describes DF as “higher-fat”, “higher-sugar” and “low energy density” but does not quantify these descriptors. For the purposes of the toolkit, DF inclusion criteria were defined based on the nutrient composition in the Modelling System:

Low fibre (≤10 g/100 g)
High fibre (>10 g/100 g)
Low fat (≤15 g/100 g)
High fat (>15 g/100 g)
High sugar (>30 g sugar; >35 g sugar if contains fruit per 100 g)
High sodium (>1000 mg/100 g)

All foods and beverages explicitly mentioned in any of the ADG documents as discretionary were automatically included as DF (*i.e.*, fruit drinks, honey, bacon, meat pies, cakes, chocolate, ice cream, muesli bar, and all alcoholic beverages). Only foods that were not part of the Five Food groups, or core foods, were assessed. Considering the cut-offs in the Modelling System, other popular foods and beverages were analyzed for nutrient composition using NUTTAB2010 [19], the most recent reference database from Food Standards Australia New Zealand at the time of this analysis, that contains data on the nutrient content of Australian foods. Their inclusion in the DF list was determined using the Modelling System cut-offs and by popularity of consumption. For example, banana cake was chosen over black forest cake and a croissant was chosen over apple strudel. Total fat, saturated fat, total sugars and sodium per 100 g were recorded for each DF under each category. Foods and beverages high in sugars were assessed by a dietitian to determine if they were typical sources of added sugars; those high in fat were assessed for their saturated fat content.

The DF list was then organized into consumer-friendly categories that reflect the current food supply. Food group terminology from NUTTAB2010 and online supermarket categories were used. A total of 10 DF groups and 72 foods and beverages were included. Examples of foods and beverages within each group are presented in Table 4.

**Table 4:** DF groups and examples of foods and beverages within each group.

Discretionary Food (DF) Group	Foods and Beverages	
Deli meats	Streaky bacon, fat not trimmed	Salami
	Ham, fat not trimmed (Prosciutto)	
	Sliced luncheon meats (e.g., Mortadella)	Sausages (including continental and frankfurter)
Take-away and frozen foods	Meat pie	Hamburger
	Sausage roll	Hot chips
	Dim sim (dumplings), spring roll, Pizza	Creamy style quiche (e.g., quiche Lorraine)
Confectionary	Chocolate bar/blocks	Lollies/sugar confectionary
	Chocolate coated bars or wafers	Rocky road
	Chocolate coated fruit/nuts	Jelly snakes
Dessert foods	Chocolate pudding	Ice cream (regular fat)
	Chocolate mousse	Ice blocks (fruit‑juice based)
	Pavlova	Ice block, chocolate coated, cream-based
Sweet biscuits and bars	Plain biscuits	Cream‑filled biscuits
		Muesli or breakfast bars
	Chocolate coated biscuits	Puffed rice bars
Bakery products	Lamingtons	Sweet muffins
	Sponge cake (cream and jam-filled)	Doughnuts
	Chocolate cake with icing	Slices (e.g., caramel/chocolate/coconut)
	Banana cake	Fudge
	Cheesecake	
	Fruit cake/pie	Cupcakes
Savoury foods and snacks	Potato crisps	Savoury flavoured crackers
	Corn chips	Buttered popcorn
	Cheese rings	Cracker/pea mixes and noodle snacks
Sauces, syrups, spreads and dips	Tomato sauce or other (e.g., sweet chilli, BBQ)	Jams
	Cream salad dressings	Butter
	Chocolate hazelnut spreads	Cream (e.g., whipped, thickened *etc.*)
	Honey/maple syrups/golden syrup	Creamy dips (e.g., French onion)
Alcoholic beverages	Full strength beer (5%)	Spirits
	Mid‑strength beer (3.5%) and light beer (2.1%)	Cocktails
	Red wine	Alcopop
	White wine/sparkling white wine	Cider
Non-alcoholic beverages	Sports drink	Fruit drink/iced tea
	Vitamin water	Cordial/diet cordial
	Soft drink/diet soft drink	Energy drink

As serve size remains consistent and portion size changes, the tool was developed to reflect real portion sizes as consumed. For each DF, a typical serve size was determined using the portion sizes of those foods as depicted by Food Works Professional [20] and in their corresponding consumer smartphone application Easy Diet Diary [21]—Two dietary recall tools commonly used by dietitians and nutritionists in Australia, as well as consumers.

Portion size for each DF in each category was recorded in grams, and the energy (kJ) content for all DF were calculated. In addition, a small, medium and large portion size for two specific DF (hot chips and muffin) were calculated to depict the effect that portion has on number of DF serves.

The kilojoule content of each DF portion was divided by 600 kJ and mathematically rounded to the nearest 0.5 to convert it to a serve. For example, chocolate pudding has 1272 kJ/100 g; one portion equals one “regular serve” or 90 g. There are 1145 kJ per regular serve; divided by 600 kJ equals 1.91, or 2 DF serves.

### 2.5. Healthcare Professional Guide

The healthcare professional guide details the ADG guideline 3 and explains additional serves. Included in the toolkit is a step-by-step guide detailing how to use the educational materials. It describes the RR and client brochure, suggests steps on how to use the toolkit and provides an example case study.

## 3. Results

The Additional Serves toolkit consists of three resources:

1. The Additional Serves Ready Reckoner
2. A consumer brochure describing “*How discretionary foods fit into a healthy diet*”
3. A *Health Professional Guide to Additional Serves Resources*

**Figure 1:**
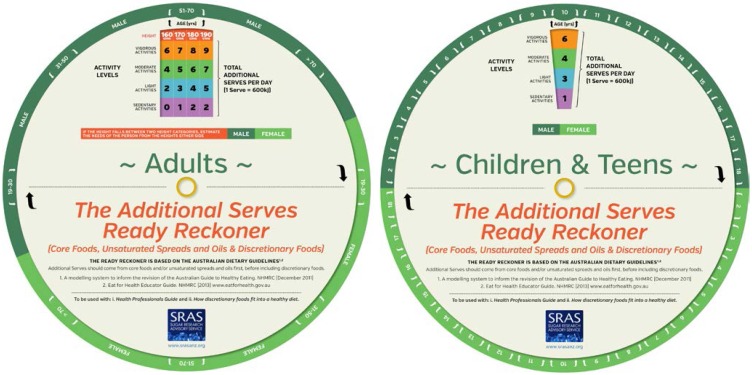
The additional serves ready reckoner.

The *Additional Serves Ready Reckoner (RR)* is used to estimate the additional serves allowance (Figure 1). Applying gender, age, height and physical activity level, the RR readily provides a maximum daily number of additional serves that can be selected from the Five Foods groups, or core foods, unsaturated fats/oils/spreads and/or DF.

A separate RR was developed for adults and for children and adolescents. When using the RR, if a client falls between two height bands, then the serves can be estimated as a value between the two corresponding values. Additional serves are recommended to meet additional energy requirements only for people who are taller or more physically active. Additional serves, and therefore a DF allowance, are not recommended for those who are overweight or who fall in the shortest, least active category. Children and adolescents who are overweight or obese are encouraged to adhere to the Foundation Diets and avoid additional serves in order to maintain body weight while the child grows in height, thus “normalizing” BMI for age.

The physical activity measure of the RR is reflective of occupation, or usual daily physical activity, rather than planned physical activity. Thus, an individual could step up to the next physical activity category if they exercise 30 to 60 min, 4 to 5 times a week.

**Figure 2:**
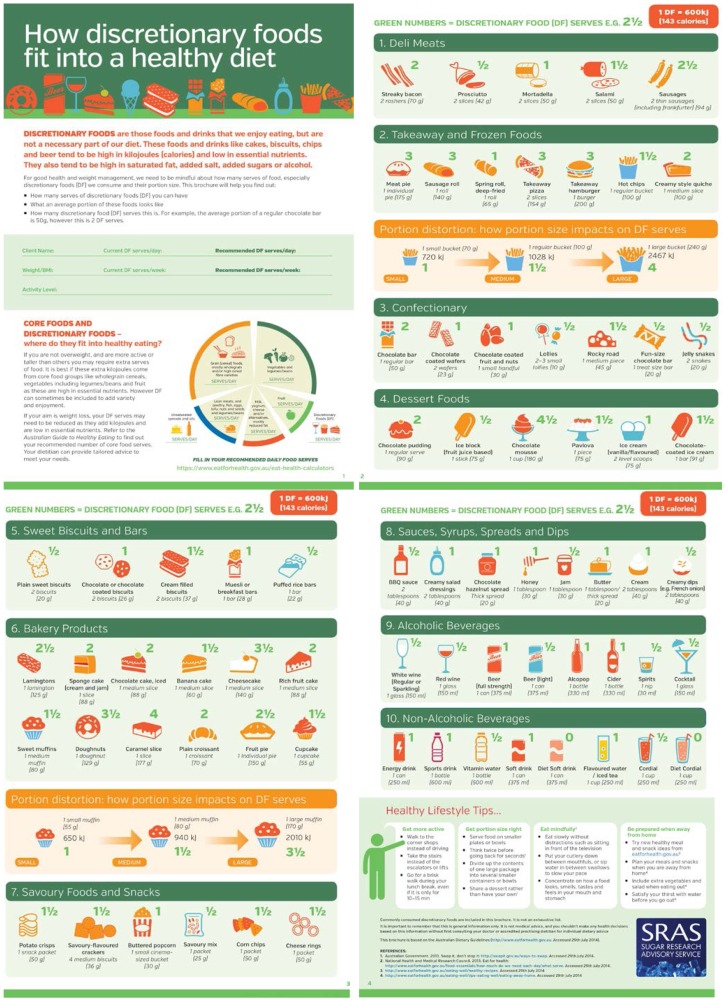
Consumer brochure.

To accompany the RR, a consumer brochure describing *“How discretionary foods fit into a healthy diet”* was developed to assist consumers in understanding how many DF serves are contained in common portions of DF (Figure 2). The consumer brochure was designed as an interactive educational tool and can be personalized to help encourage consumers to assess their DF intake relative to their maximum allowance. The brochure depicts 72 foods and beverages in 10 categories and states the equivalent number of DF for a typical portion size. It includes a descriptor of each DF and its equivalent portion size in grams or mL and using common household measures (e.g., 1 can/375 mL; 2–3 small/10 g; 1 regular bucket/100 g). Two sections called “*Portion distortion: How portion size impacts on DF serves*” illustrate the food/beverage, energy content (kJ) and total number of DF serves for that portion size. For example, hot chips are illustrated as small (1 small bucket—70 g; 720 kJ), medium (1 regular bucket/100 g; 1028 kJ) and large (1 large bucket—240 g; 2467 kJ); corresponding to 1, 1½ and 4 DF serves. The consumer brochure includes “*Healthy Lifestyle Tips”*, which outline four key recommendations from the Eat for Health website [18] on how to “*Get more active”*, “*Get portion size right*” “*Eat mindfully*” and “*Be prepared when away from home*”.

**Figure 3:**
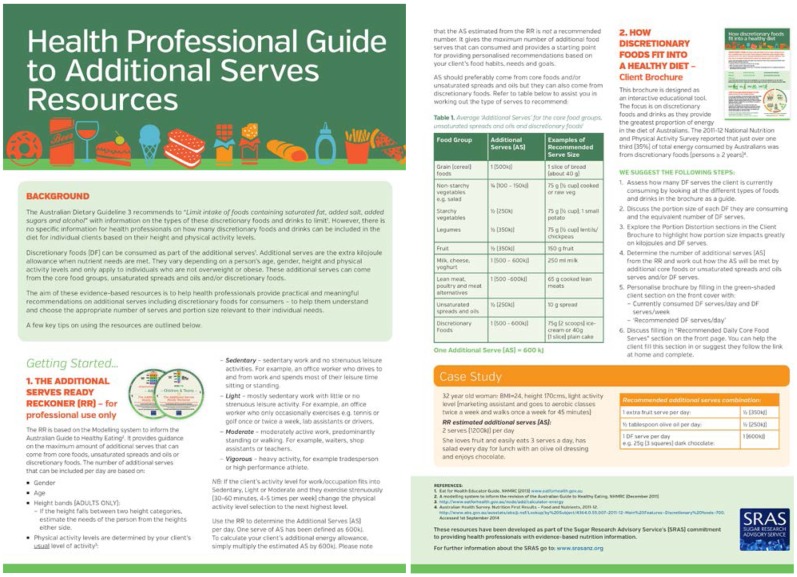
Health professional guide to additional serves resources.

The *Health Professional Guide to Additional Serves Resources* was developed to assist dietitians in using the RR and the accompanying consumer brochure to translate the guideline “to limit” into practical and ersonalized advice (Figure 3). It includes information on guideline 3 of the ADG, explains how to use the additional serves resources and provides an example as a case study.

### 3.1. How to Use the Additional Serves Resources

Applying gender, age, height and physical activity level to the RR results in a number ranging from 0 to 11. The number is not a recommendation, rather it provides the maximum number of additional serves that can be included in the diet per day providing a starting point for personalized dietary recommendations based on an individual food habits, needs and goals. Additional serves should preferably be consumed as core foods and/or unsaturated fats/oils/spreads over DF [10].

The following is the step-by-step process suggested to dietitians when using the RR:

1. Assess how many and how much (*i.e.*, portion size) DF serves the client is currently consuming.
2. Use the RR to calculate number of additional serves based on age, height and physical activity level. Depending on the client, the number of DF serves can be averaged for those individuals who fall between two height bands. DF serves are not recommended for those who are overweight or obese.
3. Make recommendations of how the additional serves can be met using a combination of core foods, unsaturated fats/oils/spreads and/or DF serves. It is important to highlight how portion size impacts greatly on kilojoules and DF serves.

### 3.2. Case Study

A 35-year-old woman, 170 cm tall and very active (*i.e.*, high activity) requires an extra 4100 kJ, or 7 additional serves, per day to meet her dietary needs. These additional serves can be consumed through intake of core foods, unsaturated fats/oils/spreads and/or DF. For example, 4 × grains (3.5 serves), 2 × fruit (1 serve), 1 × starchy vegetable (0.5 serve), 1 × legume (0.5 serve), 1 × salad vegetable (0.5 serve) and 1 × DF (1 serve) or any other preferred combination at the discretion of the healthcare professional and/or consumer based on dietary patterns and preferences.

## 4. Discussion

A food-based educational toolkit that helps translates guideline 3 of the ADG into practical, actionable advice was developed. The toolkit includes a RR, a consumer brochure and a healthcare professional guide, and is used to estimate the additional serves allowance of an individual accounting for their gender, age, height and physical activity level. To our knowledge, this is the first tool of its kind; designed to assist dietitians start the dialogue and offer personalized, food‑based advice on incorporating DF into the diet. It is intended that providing realistic targets to help consumers understand how to include DF into their diet will support behaviour change. With approximately 60% of Australians overweight and DF contributing 35% and 39% of the total daily energy intake of adults and children and adolescents, respectively, consumers need practical advice on how to translate “limit”, “avoid”, “reduce”, or consume these foods and beverages “sometimes” or “occasionally”.

DF have a place within the diets of Australians; however, when DF displace nutrient-rich core foods they can affect the nutrient profile of the diet and influence weight. Energy from nutrient-poor foods such as cookies, candy and sugar-sweetened beverages was shown to be more closely related to body mass index (kg/m^2^) than fruit and vegetable consumption or physical activity suggesting these types of foods and beverages are important targets for obesity prevention campaigns [22]. In a cluster analysis using diet history data from two clinical weight loss trials, correcting exposure to DF was shown to be key to successful weight loss in individuals with a dietary pattern characterised by non-core foods and drinks, higher- and medium-fat dairy foods, fatty meats and alcohol. Subjects who reportedly consumed larger amounts of these foods and beverages at baseline were able to alter their dietary pattern more successfully to achieve an energy deficit [23]. Therefore, adequately quantifying DF and ensuring advice is given specifically regarding these foods within the diet prescription may increase awareness of appropriate food choices and portion size, and assist with compliance [23].

The toolkit not only determines how many additional serves can be included as part of a well-balanced diet but also provides guidance on where those additional serves should come from (*i.e.*, from one of the five core food groups, from unsaturated fats/oils/spreads or from DF). Offering individuals more choice can empower them to make decisions about their diet that work for them and foster compliance. While the ADG provide general recommendations for a population, the RR tailors advice to the individual that may facilitate dietary change.

FBDG worldwide include implementation strategies that attempt to translate recommendations into consumer-friendly advice. The ADG are complemented by a website that provides resources to support educators and consumers with implementing the recommendations, advice and tips on eating well, and calculators that estimate energy and nutrient needs and the number of serves to meet recommendations [18]. In the United States, federal agencies, regional and state offices, food assistance programs, food and health organization and local community educators communicate messages and implement guidance based on the 2010 dietary guidelines for Americans. Resources to help communicate the dietary guidelines, including consumer messages, tools, and educational materials, are also available at various websites, including ChooseMyPlate.gov and healthfinder.gov. Consumers are offered daily food plans, a BMI calculator and tips on healthy eating and on how to reduce certain foods and beverages (e.g., “Compare sodium in foods like soup, bread, and frozen meals and choose the foods with lower numbers.” “Drink water instead of sugary drinks”) [24]. The Chilean implementation strategy included the development of written educational materials and training for health professionals on using and communicating the dietary guidelines to the public [2]. The National Institute of Nutrition in India produced booklets, leaflets, posters and folders with emphasis on pictorial representation of the messages to coincide with the release of their food‑based dietary guidelines [16]. And the Ministry of Health in Malaysia organized a series of advocacy and training workshops for nutritionists and other health care professionals, as well as the food industry, in an effort to widely disseminate their guidelines. Activities included provision of educational materials, seminars and workshops, as well as road shows and exhibitions at the community level [25].

Despite the well-recognised importance of translating dietary guidelines into practical advice for consumers, there is little research conducted in this area. Instead research considers population compliance to dietary recommendations rather than developing strategies to assist behaviour change [16,26,27,28,29,30]. “The need for translating the evidence into real behaviour change has never been greater, as has the need for appropriate communications to the public [31].”

The toolkit not only provides consumers with a personalized target intake for DF but it may assist with consumer education on how portion size and physical activity influence additional energy allowance. Advocating portion-control can be an effective strategy for weight loss. Obese adults were more likely to achieve and maintain meaningful weight loss when limiting portion sizes [32]. And obese children found a portion‑controlled diet easier to follow compared with a reduced‑carbohydrate diet [33].

Physical activity has also been shown as a successful intervention for weight loss and weight maintenance [4,34]. As age, gender and height are independent variables in the toolkit, it is physical activity level that has the greatest impact on the additional serves allowance. Although the physical activity category is based on occupation, the fact that a higher physical activity category results in a higher additional serves allowance may be one way of encouraging consumers to exercise more in order to include DF into their diets.

While every attempt was made to ensure accuracy in developing the RR, modelling of dietary intake has inherent limitations. All values were rounded, including height estimation, the additional energy allowance values given by the ADG, the kilojoule content of DF and the values for serves. Despite being derived from estimates, the RR provides consumers with a numerical understanding of the total additional serves that will fit into their diet per day based on variables relevant to them. Importantly, throughout development of the RR, it was tested on a group of 6 dietitians and advice was sought from both the Dietitians Association of Australia and the Australian Government Department of Health.

As with any implementation strategy, there is a need for the tool to be evaluated for effectiveness among dietitians and to be assessed for its impact on the actual eating behaviour in the general population. Efficacy cannot be determined in the absence of monitoring and critical evaluation. Further research is needed to evaluate the effectiveness of the resource. Ideally, this would include some measure of dietary change by the individual.

## 5. Conclusions

In conclusion, this toolkit was designed to assist dietitians and consumers to translate guideline 3 of the ADG and develop a personalized approach to include DF as part of the diet.

## Appendix

**Table A1:** Additional serves by age, gender and physical activity levels—Boys.

Age (Years)	Very Sedentary (PAL 1.4)		Sedentary		Moderate		Vigorous (PAL 2.0)	
	Per Day	Per Week	Per Day	Per Week	Per Day	Per Week	Per Day	Per Week
2	0	0	1	7	2	14	3	21
3	1	7	1	8	2	16	3	23
4	0	0	1	8	2	16	4	25
5	1	4	2	12	3	21	4	30
6	1	7	2	16	4	26	5	35
7	2	11	3	21	4	30	6	41
8	2	14	4	25	5	35	7	47
9	0	0	2	12	3	23	5	34
10	1	6	3	18	4	29	6	42
11	2	11	3	23	5	36	7	49
12	0	0	2	13	4	27	6	40
13	1	6	3	21	5	35	7	49
14	0	0	2	15	4	30	7	46
15	1	7	3	22	6	39	8	55
16	2	12	4	29	7	46	9	63
17	2	16	5	34	7	51	10	69
18	3	19	5	37	8	55	11	74

**Table A2:** Additional serves by age, gender and physical activity levels—Girls.

Age (Years)	Very Sedentary (PAL 1.4)		Sedentary		Moderate		Vigorous (PAL 2.0)	
	Per Day	Per Week	Per Day	Per Week	Per Day	Per Week	Per Day	Per Week
2	0	0	1	7	2	14	3	21
3	1	7	1	9	2	15	3	22
4	0	0	1	8	2	15	3	23
5	1	4	2	11	3	20	4	28
6	1	7	2	15	4	25	5	33
7	2	11	3	20	4	29	6	39
8	2	14	4	25	5	34	6	44
9	0	0	2	11	3	21	5	32
10	1	4	2	14	4	25	5	36
11	1	7	3	19	4	30	6	42
12	0	0	2	13	4	25	5	37
13	1	5	3	18	4	30	6	43
14	0	0	2	13	4	26	6	40
15	0	1	2	15	4	29	6	42
16	1	4	2	16	4	30	6	44
17	1	4	3	18	5	32	7	46
18	1	5	3	19	5	33	7	47

**Table A3:** Additional serves by age, gender and physical activity levels—Adult males.

Age (years)	Height (cm)	Very Sedentary (PAL 1.4)		Sedentary		Moderate		Vigorous (PAL 2.0)	
		Per Day	Per Week	Per Day	Per Week	Per Day	Per Week	Per Day	Per Week
19–30	160	0	0	2	15	4	30	7	46
	170	1	8	3	23	6	40	8	56
	180	2	15	5	33	7	50	10	68
	190	4	25	6	42	9	61	11	79
31–50	160	0	0	2	15	4	29	6	44
	170	1	6	3	21	5	37	8	53
	180	2	12	4	28	6	44	9	62
	190	3	18	5	35	8	53	10	70
51–70	160	0	0	2	13	4	26	6	39
	170	1	5	3	19	5	34	7	48
	180	2	11	4	26	6	41	8	57
	190	2	16	5	34	7	49	9	65
>70	160	0	0	2	12	4	25	5	36
	170	1	6	3	19	5	32	7	46
	180	2	12	4	26	6	40	8	54
	190	3	19	5	34	7	48	9	63

**Table A4:** Additional serves by age, gender and physical activity levels—Adult females.

Age (Years)	Height (cm)	Very Sedentary (PAL 1.4)		Sedentary		Moderate		Vigorous (PAL 2.0)	
		Per Day	Per Week	Per Day	Per Week	Per Day	Per Week	Per Day	Per Week
19–30	150	0	0	2	13	4	25	5	36
	160	1	7	3	20	5	33	7	47
	170	2	15	4	29	6	43	8	57
	180	3	22	5	37	8	53	10	68
31–50	150	0	0	2	13	4	25	5	36
	160	1	4	2	16	4	29	6	42
	170	1	8	3	21	5	35	7	48
	180	2	12	4	26	6	40	8	54
51–70	150	0	0	2	12	3	23	5	34
	160	1	5	2	16	4	28	6	41
>70	170	1	8	3	21	5	34	6	44
	180	2	13	4	26	6	40	8	53
	150	0	0	2	11	3	21	5	33
	160	1	5	2	15	4	27	6	39
	170	1	8	3	21	5	33	6	44
	180	2	14	4	26	6	39	7	51
