# Addendum: Fayet-Moore F., Pearson, S. Interpreting the Australian Dietary Guideline to “Limit” into Practical and Personalised Advice. *Nutrients* 2015, *7*, 2026–2043

**DOI:** 10.3390/nu7064828

**Published:** 2015-06-15

**Authors:** Flavia Fayet-Moore, Suzanne Pearson

**Affiliations:** Nutrition Research Australia, Level 13 167 Macquarie St, Sydney 2000, Australia; E-Mail: suzanne@nraus.com

The published article’s Acknowledgement Section was incomplete [1].

A research grant from the Australian Sugar Alliance was provided for this work. Appetite Communications assisted with the development of the resource into a consumer-friendly toolkit.

The authors wish to, therefore, revise the Acknowledgment Section and change it to:

A research grant from the Australian Sugar Alliance was provided for this work. Appetite Communications developed the toolkit concept, was actively involved in the research and content development and managed the production of the toolkit.
